# The Association Between Solid Fuel Use and Lower Urinary Tract Symptoms Suggestive of Benign Prostatic Hyperplasia in Sichuan, China: Cross-Sectional Study

**DOI:** 10.2196/53673

**Published:** 2024-10-31

**Authors:** Qiming Yuan, Xianghong Zhou, Li Ma, Boyu Cai, Zilong Zhang, Linghui Deng, Dan Hu, Zhongyuan Jiang, Mingda Wang, Qiang Wei, Shi Qiu

**Affiliations:** 1 Department of Urology, Institute of Urology, National Clinical Research Center for Geriatrics and Center of Biomedical Big Data, West China Hospital of Sichuan University, Chengdu, China; 2 Institute of Hospital Management of West China Hospital/West China School of Nursing, Sichuan University, Chengdu, China; 3 Neurodegenerative Disorders Lab, Laboratories for Translational Research, Ente Ospedaliero Cantonale, Bellinzona, Switzerland; 4 Clinical Research Department, West China Hospital, Sichuan University, Chengdu, China

**Keywords:** benign prostatic hyperplasia, lower urinary tract symptoms, solid fuel, household air pollution, China, male, cohort study, prostate, aging, smoking, alcohol

## Abstract

**Background:**

Benign prostatic hyperplasia (BPH) is a global age-related disease. It has been reported that over half of the Chinese male population aged 70 years or older are experiencing BPH. Solid fuel, which is the major source of household air pollution, has been reportedly associated with several adverse events, including sex hormone disorders. Due to the certain relationship between sex hormone levels and prostate disease, the relationship between solid fuel use and lower urinary tract symptoms (LUTSs) suggestive of BPH (LUTS/BPH) deserves further exploration.

**Objective:**

This study mainly aimed to investigate the association between solid fuel use and LUTS/BPH.

**Methods:**

The data used in this study were obtained from the West China Natural Population Cohort Study. Household energy sources were assessed using questionnaires. LUTS/BPH was evaluated based on participant self-reports. We performed propensity score matching (PSM) to reduce the influence of bias and unmeasured confounders. The odds ratio (OR) and 95% CI of LUTS/BPH for the solid fuel group compared with the clean fuel group were calculated. We also conducted stratified analyses based on BMI, metabolic syndrome, waist to hip ratio, drinking status, smoking status, and age.

**Results:**

A total of 5463 participants were included in this study, including 399 solid fuel users and 5064 clean fuel users. After PSM, the solid fuel group included 354 participants, while the clean fuel group included 701 participants. Solid fuel use was positively correlated with LUTS/BPH before and after PSM (OR 1.68, 95% CI 1.31‐2.15 and OR 1.81, 95% CI 1.35‐2.44, respectively). In stratified analyses, the OR of the nonsmoking group was higher than that of the smoking group (OR 2.56, 95% CI 1.56‐4.20 and OR 1.47, 95% CI 0.99‐2.18, respectively). Similarly, the OR of the nondrinking group was higher than that of the drinking group (OR 2.70, 95% CI 1.46‐4.99 and OR 1.48, 95% CI 1.01‐2.17, respectively).

**Conclusions:**

A positive correlation between solid fuel use and LUTS/BPH was observed. The results suggest that improving fuel structure for household cooking and other household needs can possibly help reduce the risk of LUTS/BPH.

## Introduction

Benign prostatic hyperplasia (BPH) is the progressive enlargement of the prostate gland as a result of the nonmalignant proliferation of epithelial and stromal cells [1,2]. BPH is an age-related disease. It has been reported that over half of the Chinese male population aged 70 years or older are experiencing BPH [3]. The pathogenesis of BPH remains incompletely understood despite its high prevalence, as multiple factors are involved in the process [4]. Except for aging, several studies have attempted to identify other risk factors for BPH, including androgen and estrogen levels, cigarette and alcohol consumption, sexual activity, and socioeconomic factors [5,6]. BPH mainly presents as lower urinary tract symptoms (LUTSs), including symptoms related to bladder outlet obstruction, impaired bladder compliance, and bladder overactivity [7]. With the arrival of an aging population, identifying the potential risk factors for LUTS/BPH associated with daily life is vital to achieve healthy aging and improve the quality of life.

According to data from the Global Burden of Disease 2017 project, air pollution is the greatest environmental risk factor for mortality [8]. With the aging of the population, the amount of time individuals spend in their houses has significantly increased, and household air pollution (HAP) has attracted increasing attention. It has been reported that HAP was responsible for 3.5 million deaths in 2010 [9], and solid fuels, including biomass and coal, are the largest source of HAP. Approximately half of the population in developing countries still uses solid fuels for household needs, such as cooking [10], and solid fuels remain widely used in Chinese households in recent years [11]. Various substantial toxic pollutants can be discharged during the combustion of solid fuels, including nitrogen dioxide, carbon monoxide, particulate matter 2.5, particulate matter 10, and several carcinogenic substances [12]. Studies have proven that solid fuel use is correlated to several adverse events, including lung cancer [13], cardiovascular diseases [14], arthritis events [15], high blood pressure [16], and cognitive impairment [17].

Exposure to air pollution is clearly associated with sexual hormone disruption, further resulting in reproductive toxicity [18]. The prostate gland, which is physiologically under the control of sexual hormones, is particularly sensitive to air pollution. However, little is known about the relationship between HAP and LUTS/BPH, especially the relationship between solid fuel use and LUTS/BPH. To fill this knowledge gap, we examined the data from the West China Natural Population Cohort Study (WCNPCS), aiming to clarify whether solid fuel use is associated with LUTS/BPH.

## Methods

### Study Participants

**Figure 1.**
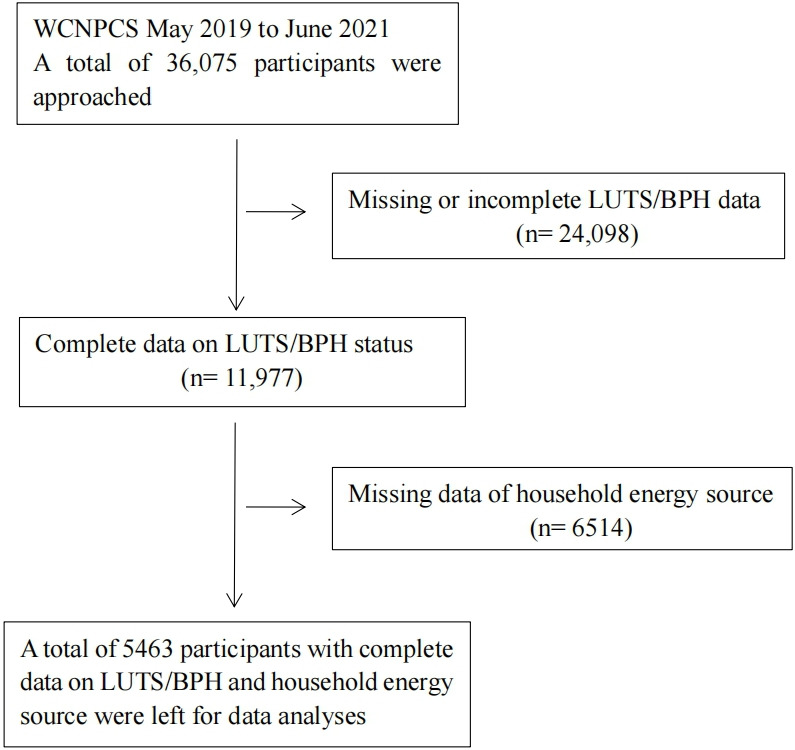
Flowchart describing the selection of participants. BPH: benign prostatic hyperplasia; LUTS: lower urinary tract symptom; WCNPCS: West China Natural Population Cohort Study.

The WCNPCS is an ongoing prospective cohort study mainly performed in Sichuan province, the most populous province in Western China [19]. The WCNPCS collected various data on the community population in Western China and evaluated their health status, aiming to establish a large-scale prospective follow-up natural population cohort. The participants of the WCNPCS were drawn from the habitual adult residents of the cooperative communities by sequential cluster sampling. Trained full-time staff conducted face-to-face questionnaires, physical examinations, collection of biological specimens, and special examinations on respondents. Cross-sectional data from a total of 36,075 participants were obtained in this wave of WCNPCS participants. The detailed procedure of the study inclusion and exclusion is presented in Figure 1.

### Ethical Considerations

This study was performed in accordance with the Helsinki Declaration. The study was approved by the ethics committee of West China Hospital of Sichuan University (2020‐1700) and registered in the China Clinical Trial Registration Center (ChiCTR1900024623). Participants were voluntarily recruited without compensation. Before the survey, each participant provided and signed informed consent. All the study data were anonymous.

### Household Energy Source

Household energy sources used in cooking were evaluated using a questionnaire. Participants who answered coal, briquettes, firewood, or charcoal were viewed as solid fuel users, and those who answered other fuel, such as natural gas, were defined as clean fuel users. Simultaneously, we asked the participants if they were frequently involved in cooking.

### LUTS/BPH Assessment

LUTS/BPH was assessed based on participant self-reports, which were frequently performed in other studies [4,20,21]. The question “Have you ever been diagnosed with prostate hyperplasia?” determined BPH. BPH-related LUTSs, including dysuria, increasing nocturia, and urinary incontinence, were assessed after explaining the symptoms to all participants. Participants who answered a clear “yes” during the survey were considered as having LUTS/BPH.

### Covariates

Data about demographic characteristics, socioeconomic factors, and lifestyle factors were collected using a questionnaire. The creatine level was measured using serum samples collected by trained medical personnel and analyzed via an enzymatic method in the hospital laboratory. Continuous covariates included age (years), waist to hip ratio (WHR), BMI (kg/m^2^), Patient Health Questionnaire-9, Pittsburgh Sleep Quality Index, Generalized Anxiety Disorder-7, and creatine level (μmol/L). Categorical covariates included smoking status (current, occasionally, ever, or never), drinking status (yes, ever, or no), educational level (primary school, junior school, high school, college, or graduate), marital status (married, unmarried, divorced, separated, or widowed), tea intake (no, 1‐2 times per week, 3‐5 times per week, or >5 times per week), coffee intake (no, 1‐2 times per week, 3‐5 times per week, or >5 times per week), physical activity (inactive, not sufficient, or sufficient), chronic heart disease (yes or no), chronic obstructive pulmonary disease (yes or no), diabetes mellitus (yes, prediabetes, or no), hypertension (yes or no), cancer (yes or no), indoor ventilation (yes or never), and current cooking status (frequent or not frequent).

### Statistical Analysis

Baseline characteristics between the clean fuel and solid fuel groups were compared. Continuous variables were presented as means (SDs), and the Kruskal-Wallis rank sum test was performed to evaluate differences. Categorical variables were presented as the frequency and its proportion. A 2-tailed chi-square test or Fisher exact test was performed to estimate differences in categorical variables. Propensity score matching (PSM) was performed to reduce selection bias, of which the propensity scores were reckoned by logistic regression. The detailed PSM information is presented in Table S1 in Multimedia Appendix 1. Moreover, baseline characteristics between the 2 groups following PSM were compared. To calculate the odds ratio (OR) and 95% CI of LUTS/BPH for the solid fuel group compared with the clean fuel group before and after matching, unadjusted and adjusted logistic regression models were performed. The fully adjusted model was adjusted for age; BMI; WHR; creatine level; smoking status; drinking status; educational level; marital status; tea intake; coffee intake; physical activity; indoor ventilation; and history of chronic obstructive pulmonary disease, chronic heart disease, diabetes mellitus, hypertension, and cancer. Considering that chronic inflammation plays an important role in the onset of BPH, subgroup analyses based on relevant factors were performed after PSM, including BMI, metabolic syndrome, WHR, drinking status, and smoking status, and the interactions were tested. Metabolic syndrome indicates a clustering of the following medical conditions: central obesity (waist≥90 cm), hypertension (systolic blood pressure≥130 or diastolic blood pressure ≥85 mm Hg or treatment of hypertension history), high fasting blood glucose (fasting blood glucose≥100 mg/dL or history of type 2 diabetes), high serum triglycerides (serum triglycerides≥150 mg/dL), and low serum high-density lipoprotein (serum high-density lipoprotein<40 mg/dL) [22]. The drinking group included participants who drunk currently or previously, while the nondrinking group included those who never drank. Smokers included those who were currently smoking, and nonsmokers included those who never, ever, or occasionally smoked. Statistical analyses were performed using the statistical software package R (R Foundation for Statistical Computing). A 2-sided test was used, and a *P* value of <.05 was considered statistically significant.

## Results

A total of 5463 participants were included in this study, including 5064 clean fuel users and 399 solid fuel users. The detailed participants’ characteristics are shown in Table 1. A total of 1156 of 5064 (22.8%) clean fuel users reported LUTS/BPH, while 150 of 399 (37.6%) participants reported LUTS/BPH. The solid fuel group had a higher mean age than the clean fuel group (*P*<.001), whereas the clean fuel group had a higher BMI and creatine level (*P*<.001 and *P*=.002, respectively). Meanwhile, the mean Generalized Anxiety Disorder-7 score of clean fuel users was higher than that of solid fuel users (*P*=.04). In total, 2118 of 5064 (41.8%) clean fuel users were currently smoking, whereas 219 of 399 (54.9%) solid fuel users were currently smoking (*P*<.001). The educational level (*P*<.001), drinking status (*P*=.02), tea intake (*P*<.001), coffee intake (*P*<.001), physical activity (*P*<.001), history of hypertension (*P*<.001), indoor ventilation (*P*<.001), and current cooking status (*P*<.001) all showed significant differences between the 2 groups.

Following PSM, 1055 participants were retained. The baseline characteristics of 701 clean fuel users and 354 solid fuel users are shown in Table 2. A total of 169 of 701 (24.1%) clean fuel users reported LUTS/BPH, while 137 of 354 (38.7%) participants reported LUTS/BPH. Except for LUTS/BPH (*P*<.001), educational level (*P*=.03), and indoor ventilation (*P*<.001), the baseline characteristics of the clean fuel and solid fuel groups showed no other significant difference.

The results of logistic regression analyses of the association between solid fuel use and LUTS/BPH are presented in Table 3. In the unadjusted model, solid fuel use was positively associated with LUTS/BPH (OR 2.04, 95% CI 1.65‐2.52). The correlation remained significant after adjusting for confounding variables (OR 1.68, 95% CI 1.31‐2.15). Following PSM, the correlation between solid fuel use and LUTS/BPH was further assessed, which remained significant in both unadjusted and adjusted models (OR 2.00, 95% CI 1.52‐2.64 and OR 1.81, 95% CI 1.35‐2.44, respectively).

**Figure 2.**
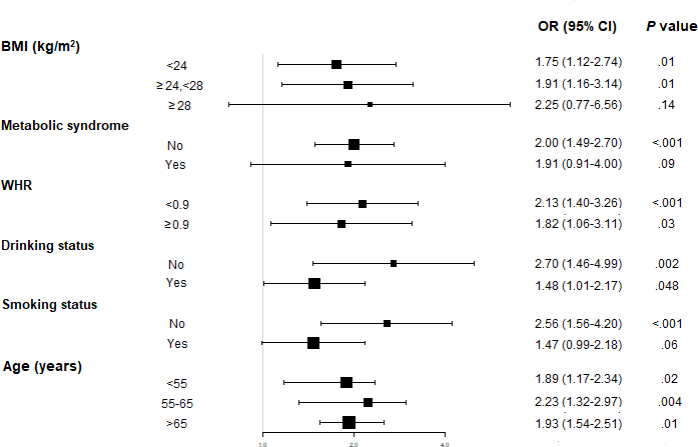
Stratified odds ratios (ORs) for lower urinary tract symptoms suggestive of benign prostatic hyperplasia between clean fuel users and solid fuel users after propensity score matching. The model was adjusted for age, BMI, waist to hip ratio (WHR), creatine, education level, marital status, smoking status, drinking status, tea intake, coffee intake, physical activity, chronic obstructive pulmonary disease, chronic heart disease, diabetes mellitus, hypertension, cancer, and indoor ventilation.

The results of the stratified analyses are shown in Figure 2. In the BMI stratified analysis, the OR of LUTS/BPH tended to increase with the rise of BMI. However, no significant association was found between the use of solid fuel and LUTS/BPH in a group with BMI≥28 kg/m^2^ (OR 2.25, 95% CI 0.77‐6.56), and the interaction was not significant (*P* for interaction=.62). Drinking status and smoking status showed significant interactions to the association between solid fuel use and LUTS/BPH (*P* for interaction=.048 and *P* for interaction=.02 respectively). Both the nondrinking group and the drinking group showed a significant association between solid fuel use and LUTS/BPH, while the OR of the nondrinking group was higher (OR 2.70, 95% CI 1.46‐4.99 and OR 1.48, 95% CI 1.01‐2.17, respectively). In the nonsmoking group, the use of solid fuel was significantly associated with LUTS/BPH (OR 2.56, 95% CI 1.56‐4.20); while in the smoking group, the association was not significant (OR 1.47, 95% CI 0.99‐2.18).

**Table 1.** Baseline characteristics of 5463 participants.

	Clean fuel (n=5064)	Solid fuel (n=399)	*P* value
Age (years), mean (SD)	56.18 (11.91)	62.20 (11.350	<.001
BMI (kg/m^2^), mean (SD)	25.08 (3.24)	24.39 (3.48)	<.001
WHR^a^, mean (SD)	0.88 (0.06)	0.89 (0.06)	.07
PSQI^b^, mean (SD)	5.75 (3.13)	5.67 (3.24)	.70
PHQ-9^c^, mean (SD)	0.77 (1.94)	0.59 (1.77)	.15
GAD-7^d^, mean (SD)	0.74 (2.08)	0.62 (2.00)	.04
Creatine (μmol/L), mean (SD)	73.74 (24.96)	69.74 (24.20)	.002
**Educational level, n (%)**			<.001
Primary school	1016 (26.3)	213 (57.1)	
Junior school	1527 (39.5)	138 (37)	
High school	725 (18.7)	19 (5.1)	
College	595 (15.4)	3 (0.8)	
Graduate	5 (0.1)	0 (0)	
**Marital status, n (%)**			.10
Married	4747 (93.8)	362 (90.7)	
Unmarried	73 (1.4)	12 (3)	
Divorced	95 (1.9)	10 (2.5)	
Separation	18 (0.4)	2 (0.5)	
Widowed	130 (2.6)	13 (3.3)	
**Smoking status, n (%)**			<.001
Current	2118 (41.8)	219 (54.9)	
Occasionally	170 (3.4)	8 (2)	
Never	2160 (42.7)	124 (31.1)	
Ever	616 (12.2)	48 (12)	
**Drinking status, n (%)**			.02
Yes	2551 (50.4)	206 (51.6)	
No	2183 (43.1)	154 (38.6)	
Ever	329 (6.5)	39 (9.8)	
**Tea, n (%)**			<.001
No	1795 (35.5)	139 (34.8)	
1‐2 times per week	759 (15)	40 (10)	
3‐5 times per week	423 (8.4)	19 (4.8)	
>5 times per week	2086 (41.2)	201 (50.4)	
**Coffee, n (%)**			<.001
No	4649 (91.8)	392 (98.3)	
1‐2 times per week	351 (6.9)	6 (1.5)	
3‐5 times per week	37 (0.7)	1 (0.3)	
>5 times per week	26 (0.5)	0 (0)	
**Physical activity, n (%)**			<.001
Inactive	1748 (34.5)	225 (56.4	
Not sufficient	728 (14.4)	39 (9.8)	
Sufficient	2588 (51.1)	135 (22.8)	
**Chronic obstructive pulmonary disease, n (%)**			.96
No	4871 (96.2)	384 (96.2)	
Yes	193 (3.8)	15 (3.8)	
**Chronic heart disease, n (%)**			.49
No	4977 (98.3)	394 (98.7)	
Yes	87 (1.7)	5 (1.3)	
**Diabetes mellitus, n (%)**			.45
No	4656 (92.3)	375 (94)	
Yes	325 (6.5)	21 (5.3)	
Prediabetes	61 (1.2)	3 (0.8)	
**Hypertension, n (%)**			<.001
No	2536 (50.1)	164 (41.1)	
Yes	2528 (49.9)	235 (58.9)	
**Cancer, n (%)**			.81
No	5017 (99.1)	396 (99.2)	
Yes	44 (0.9)	3 (0.8)	
**Indoor ventilation, n (%)**			<.001
Yes	4719 (93.4)	261 (65.4)	
Never	333 (6.6)	138 (34.6)	
**Current cooking status, n (%)**			<.001
Never	4696 (92.8)	393 (98.5)	
Frequent	367 (7.2)	6 (1.5)	
**LUTS** ^e^ **/BPH** ^f^ **, n (%)**			<.001
No	3908 (77.2)	249 (62.4)	
Yes	1156 (22.8)	150 (37.6)	

a WHR: waist to hip ratio.

b PSQI: Pittsburgh Sleep Quality Index.

c PHQ-9: Patient Health Questionnaire-9.

d GAD-7: Generalized Anxiety Disorder-7.

e LUTS: lower urinary tract symptom.

f BPH: benign prostatic hyperplasia.

**Table 2.** Baseline characteristics of 1055 participants after propensity score matching.

	Clean fuel (n=701)	Solid fuel (n=354)	*P* value
Age (years), mean (SD)	61.01 (10.06)	62.15 (11.27)	.09
BMI (kg/m^2^), mean (SD)	24.38 (3.09)	24.32 (3.49)	.77
WHR^a^, mean (SD)	0.89 (0.06)	0.89 (0.05)	.49
PSQI^b^, mean (SD)	5.54 (2.91)	5.71 (3.17)	.37
PHQ-9^c^, mean (SD)	0.46 (1.66)	0.50 (1.23)	.69
GAD-7^d^, mean (SD)	0.59 (1.96)	0.55 (1.70)	.74
Creatine (μmol/L), mean (SD)	72.82 (54.78)	69.50 (25.09)	.28
**Educational level, n (%)**			.03
Primary school	382 (54.5)	197 (55.7)	
Junior school	241 (34.4)	135 (38.1)	
High school	47 (6.7)	19 (5.4)	
College	30 (4.3)	3 (0.8)	
Graduate	1 (0.1)	0 (0)	
**Marital status, n (%)**			.18
Married	660 (94.2)	323 (91.2)	
Unmarried	7 (1)	9 (2.5)	
Divorced	11 (1.6)	10 (2.8)	
Separation	2 (0.3)	2 (0.6)	
Widowed	21 (3)	10 (2.8)	
**Smoking status, n (%)**			.27
Current	394 (56.2)	195 (55.1)	
Occasionally	27 (3.9)	8 (2.3)	
Never	214 (30.5)	107 (30.2)	
Ever	66 (9.4)	44 (12.4)	
**Drinking status, n (%)**			.38
Yes	374 (53.4)	187 (52.8)	
No	273 (38.9)	131 (37)	
Ever	54 (7.7)	36 (10.2)	
**Tea, n (%)**			.55
No	230 (32.8)	116 (32.8)	
1‐2 times per week	54 (7.7)	33 (9.3)	
3‐5 times per week	49 (7)	18 (5.1)	
>5 times per week	368 (52.5)	187 (52.8)	
**Coffee, n (%)**			.19
No	675 (96.3)	349 (98.6)	
1‐2 times per week	21 (3)	4 (1.1)	
3‐5 times per week	3 (0.4)	1 (0.3)	
>5 times per week	2 (0.3)	0 (0)	
**Physical activity, n (%)**			.54
Inactive	382 (54.5)	197 (55.7)	
Not sufficient	60 (8.6)	36 (10.2)	
Sufficient	259 (36.9)	121 (34.2)	
**Chronic obstructive pulmonary disease, n (%)**			.27
No	682 (97.3)	340 (96)	
Yes	19 (2.7)	14 (4)	
**Chronic heart disease, n (%)**			.84
No	690 (98.4)	349 (98.6)	
Yes	11 (1.6)	5 (1.4)	
**Diabetes mellitus, n (%)**			.77
No	668 (95.3)	334 (94.4)	
Yes	27 (3.9)	17 (4.8)	
Prediabetes	6 (0.9)	3 (0.8)	
**Hypertension, n (%)**			.60
No	293 (41.8)	142 (40.1)	
Yes	408 (58.2)	212 (59.9)	
**Cancer, n (%)**			.81
No	696 (99.3)	351 (99.2)	
Yes	5 (0.7)	3 (0.8)	
**Indoor ventilation, n (%)**			<.001
Yes	609 (86.9)	233 (65.8)	
Never	92 (13.1)	121 (34.2)	
**Current cooking status, n (%)**			.48
Never	700 (99.8)	354 (100)	
Frequent	1 (0.1)	0 (0)	
**LUTS** ^e^ **/BPH** ^f^ **, n (%)**			<.001
No	532 (75.9)	217 (61.3)	
Yes	169 (24.1)	137 (38.7)	

a WHR: waist to hip ratio.

b PSQI: Pittsburgh Sleep Quality Index.

c PHQ-9: Patient Health Questionnaire-9.

d GAD-7: Generalized Anxiety Disorder-7.

e LUTS: lower urinary tract symptom.

f BPH: benign prostatic hyperplasia.

**Table 3.** Odds ratios (ORs) for lower urinary tract symptoms suggestive of benign prostatic hyperplasia between clean fuel users and solid fuel users before and after propensity score matching (PSM).

Exposure		Nonadjusted		Adjusted^a^	
		OR (95% CI)	*P* value	OR (95% CI)	*P* value
**Before PSM**					
	Clean fuel	1 (—^b^)	—	1 (—)	—
	Solid fuel	2.04 (1.65-2.52)	<.001	1.68 (1.31-2.15)	<.001
**After PSM**					
	Clean fuel	1 (—)	—	1 (—)	—
	Solid fuel	2.00 (1.52-2.64)	<.001	1.81 (1.35-2.44)	<.001

a Adjusted model was adjusted for age, BMI, waist to hip ratio, creatine, education level, marital status, smoking status, drinking status, tea intake, coffee intake, physical activity, chronic obstructive pulmonary disease, chronic heart disease, diabetes mellitus, hypertension, cancer, and indoor ventilation.

b Not applicable.

## Discussion

Using data from the WCNPCS and based on a well-designed sample, the results of this study showed that using solid fuel for household needs was related to a higher risk of LUTS/BPH. The association remained significant even after PSM to simulate a randomized trial design and reduce selection bias. This study contributed to the knowledge gap on the association between solid fuel use and LUTS/BPH, particularly for individuals who lived in Western China where the prevalence of solid fuel use was high [23].

To date, few studies pay attention to the correlation between air pollution and BPH or LUTS, particularly HAP from solid fuels and LUTS/BPH. One previous study explored the correlation between 7 different air pollutants (carbon monoxide, NOx, SOx, particulate matter 10, volatile organic compounds, total suspended particles, and NH_3_) and BPH and reported that an increasing overall concentration of air pollutants could result in an increased risk of BPH [24]. However, the analyses of this study only adjusted for age, and there were other covariates that could disrupt the results. Furthermore, HAP is a complex cocktail of chemicals. Except for the abovementioned 7 types of air pollutants, there are some other components, including polycyclic aromatic hydrocarbons (PAHs), which have been linked to various adverse health outcomes [25]. The logistic regression analyses of our study were adjusted for several confounding variables, including socioeconomic covariates, daily behaviors, and previous disease status. Furthermore, we performed PSM to simulate a randomized trial design and verified our results.

The correlation between HAP from solid fuel use and LUTS/BPH can be explained by the following 2 mechanisms: sex hormones and oxidative stress. Previous studies have confirmed that androgens can participate in BPH development by directly affecting prostate tissues. One study included 93 patients with BPH and observed that patients with larger prostates had significantly higher androgen levels than those with smaller prostates [26]. Moreover, estrogens play a crucial role in BPH. It has been shown that when rats were treated with a combination of androgen and estrogen, the rate of increase in prostate weight was higher than that with androgen treatment alone [27]. PAHs, as important components of HAP, can disrupt serum sex hormone levels. Studies have shown that exposure to some types of PAHs could result in increased androgen and estrogen levels [28,29], which may further contribute to the occurrence of BPH. Oxidative stress is another possible mechanism and is defined as an imbalance in the production and detoxification of reactive nitrogen species, inducible nitric oxide, and reactive oxygen species [30]. Nitric oxide synthase activated by inducible nitric oxide synthase had greater immunostaining in the epithelial cells of a hyperplastic prostate than that in normal prostate tissue [31]. Several gases from HAP have oxidative properties, which can induce oxidative stress [32], and oxidative stress can further trigger systematic inflammation by increasing proinflammatory cytokine production [33]. The occurrence of prostatic tissue oxidative stress imbalance and inflammation can result in growth factor and inflammatory cytokine accumulation and significantly contribute to BPH [34]. In addition, systemic and prostate-specific chronic inflammation and oxidative stress are common characteristics of obesity, and obesity can promote the occurrence of LUTS/BPH through autophagy deregulation [35].

We observed that drinking status and smoking status had a significant interaction with the correlation of solid fuel use and LUTS/BPH. The stratified analysis based on drinking status and smoking status showed that the risk of LUTS/BPH increased more significantly among nondrinking participants and nonsmoking participants. It is reported that HAP from solid fuel combustion played a more important role in the occurrence of tissue inflammation for participants who did not drink or smoke [36]. One possible explanation is that participants who did not drink or smoke were more sensitive to HAP, which can cause greater damage to their health.

Although this is the first study analyzing the correlation between solid fuel use and LUTS/BPH with PSM, some limitations still existed. First, we can hardly determine the causal relationship between solid fuel use and LUTS/BPH because of the characteristics of a cross-sectional design. Second, as an observational study, although a number of covariates had been adjusted, and PSM was performed to simulate a randomized trial design, unmeasured confounding could still not be excluded. Third, the information directly reflecting previous prostatitis, sexual activity, household income, work activity, and occupational exposure is not available in the WCNPCS database, which may cause certain interference to the results. Besides, the diagnosis of LUTS/BPH was based on a self-report questionnaire, which might lead to omissions in some patients experiencing BPH with milder symptoms. Meantime, solid fuel use was also assessed through a self-report questionnaire, so we could not measure the dose and components of exposure; therefore, analyzing the association between the components of HAP and LUTS/BPH was challenging.

In conclusion, a positive correlation between solid fuel use and LUTS/BPH was noted in this study. The results suggest that improving fuel structure for household cooking and other household needs can possibly reduce the risk of LUTS/BPH.

## Supplementary material

10.2196/53673 Multimedia Appendix 1 Propensity score parameter list, baseline characteristics of participants with or without fuel information, and stratified analysis based on rural and urban participants.

## Abbreviations

BPH: benign prostatic hyperplasia
HAP: household air pollution
LUTS: lower urinary tract symptom
OR: odds ratio
PAH: polycyclic aromatic hydrocarbon
PSM: propensity score matching
WCNPCS: West China Natural Population Cohort Study
WHR: waist to hip ratio
