# Supplementary material for: The Association Between Solid Fuel Use and Lower Urinary Tract Symptoms Suggestive of Benign Prostatic Hyperplasia in Sichuan, China: Cross-Sectional Study
Source: JMIR Public Health Surveill. 2024 Oct 31;10:e53673. doi: 10.2196/53673 (PMC11542908; doi:10.2196/53673)
Supplement: Multimedia Appendix 1 [file publichealth-v10-e53673-s001.docx]

**Table S1. Propensity score parameter list.**

| The variables used in calculating the propensity matching | Age, BMI, Education, Martial status, Smoking, Drinking, Tea, Coffee, WHR, Physical activity, Chronic disease, Creatinine, Indoor Ventilation, Cooking Status | |
| --- | --- | --- |
| Propensity scoring algorithm | Logistic regression model | |
| C-statistical | 0.9211 | |
| Matching method | Greedy matching within specified caliper distances | |
| Distance metric | 0.01 | |
| Matching ratio | (Solid fuel) 1:2 (Clean fuel) | |
| Use of replacement | With replacement | |
| Matching sample size | Solid fuel: 354 cases | Total: 1055 cases |
|  | Clean fuel: 701 cases |  |

Abbreviations

BMI: body mass index; WHR: waist-to-hip ratio

**Table S2. Baseline characteristics of participants with or without fuel information.**

|  | With Fuel Information  (n=5463) | Without Fuel Information  （n=6514） | p value |
| --- | --- | --- | --- |
| **Age, year, mean (SD)** | 56.62(11.87) | 57.10(10.68) | 0.021 |
| **BMI, kg/m^2^, mean (SD)** | 25.03(3.26) | 24.95(3.59) | 0.201 |
| **WHR, mean (SD)** | 0.88(0.06) | 0.88(0.07) | 0.990 |
| **PSQI, mean (SD)** | 5.72(3.18) | 5.80(3.50) | 0.190 |
| **PHQ9, mean (SD)** | 0.71(1.85) | 0.68(1.97) | 0.391 |
| **GAD7, mean (SD)** | 0.67(1.99) | 0.62(2.21) | 0.193 |
| **Cr, umol/L, mean (SD)** | 71.85(23.57) | 72.00(26.13) | 0.741 |
| **Educational level, n (%)** |  |  | 0.045 |
| primary | 1229(22.50) | 1973(30.28) |  |
| junior | 1665(30.48) | 2371(36.40) |  |
| high | 744(13.62) | 1232(18.91) |  |
| college | 598(10.95) | 928(14.24) |  |
| graduate | 5(0.09) | 7(0.11) |  |
| **Marital status, n (%)** |  |  | 0.995 |
| married | 5109(93.52) | 6101(93.65) |  |
| unmarried | 85(1.56) | 97(1.49) |  |
| divorced | 105(1.92) | 125(1.92) |  |
| separation | 20(0.37) | 24(0.37) |  |
| widowed | 143(2.62) | 164(2.52) |  |
| **Smoking status, n (%)** |  |  | 0.165 |
| current | 2337(42,78) | 2668(40.96) |  |
| occasionally | 178(3.26) | 202(3.10) |  |
| never | 2284(41.81) | 2847(43.71) |  |
| ever | 664(12.15) | 796(12.23) |  |
| **Drinking status, n (%)** |  |  | 0.166 |
| yes | 2757(50.47) | 3298(50.63) |  |
| no | 2337(42.78) | 2721(41.77) |  |
| ever | 368(6.74) | 493(7.59) |  |
| **Tea, n (%)** |  |  | 0.171 |
| no | 1934(35.40) | 2403(36.88) |  |
| 1-2 times per week | 799(14.63) | 886(13.60) |  |
| 3-5 times per week | 442(8.09) | 494(7.58) |  |
| >5 times per week | 2287(41.86) | 2728(41.85) |  |
| **Coffee, n (%)** |  |  | 0.232 |
| no | 5041(92.28) | 5949(91.32) |  |
| 1-2 times per week | 357(6.53) | 489(7.51) |  |
| 3-5 times per week | 38(0.70) | 44(0.68) |  |
| >5 times per week | 26(0.48) | 31(0.48) |  |
| **Physical activity, n (%)** |  |  | 0.353 |
| inactive | 1973(36.12) | 2308(35.42) |  |
| not sufficient | 767(14.04) | 876(13.45) |  |
| sufficient | 2723(49.84) | 3329(51.13) |  |
| **COPD, n (%)** |  |  | 0.263 |
| no | 5255(96.19) | 6291(96.58) |  |
| yes | 208(3.81) | 222(3.42) |  |
| **CHD, n (%)** |  |  | 0.397 |
| no | 5371(98.32) | 6417(98.51) |  |
| yes | 92(1.68) | 96(1.49) |  |
| **DM, n (%)** |  |  | 0.270 |
| no | 5031(92.09) | 6072(93.23) |  |
| yes | 346(6.33) | 372(5.71) |  |
| prediabetes | 64(1.17) | 69(1.06) |  |
| **Hypertension, n (%)** |  |  | 0.394 |
| no | 2700(49.42) | 3271(50.20) |  |
| yes | 2763(50.58) | 3242(49.80) |  |
| **Cancer, n (%)** |  |  | 0.043 |
| no | 5413(99.08) | 6459(99.15) |  |
| yes | 47(0.92) | 55(0.85) |  |

Abbreviations

Cr: Creatine; BMI: body mass index; WHR: waist-to-hip ratio; PSQI: Pittsburgh sleep quality index; PHQ9: Patient Health Questionnaire-9; GAD7: Generalized Anxiety Disorde-7; COPD: chronic obstructive pulmonary disease; CHD: chronic heart disease; DM: diabetes mellitus; SD: standard deviation

**Table S3. Stratified analysis based on rural and urban participants.**

|  | exposure | Non-adjusted (OR, 95%CI) | Adjusted (OR, 95%CI) |
| --- | --- | --- | --- |
| Rural | clean fuel | 1 | 1 |
| (n=2001) | solid fuel n=242 | 2.16, (1.65, 2.82) | 1.88, (1.35, 2.01) |
|  |  | p<0.001 | p<0.001 |
| Urban | clean fuel | 1 | 1 |
| (n=3462) | solid fuel n=157 | 1.98, (1.42, 2.74) | 1.62, (1.23, 1.94) |
|  |  | p<0.001 | p<0.001 |

Adjusted model was adjusted for age, BMI, WHR, Cr, education level, marital status, smoking status, drinking status, tea intake, coffee intake, physical activity, COPD, CHD, DM, hypertension, cancer, and indoor ventilation.
